# Hypoxia sensing by hepatic stellate cells leads to VEGF-dependent angiogenesis and may contribute to accelerated liver regeneration

**DOI:** 10.1038/s41598-020-60709-9

**Published:** 2020-03-09

**Authors:** Konstantin Dirscherl, Martin Schläpfer, Birgit Roth Z’graggen, Roland H Wenger, Christa Booy, Renata Flury-Frei, Rita Fatzer, Costica Aloman, Birke Bartosch, Romain Parent, Vartan Kurtcuoglu, Diane de Zélicourt, Donat R. Spahn, Beatrice Beck Schimmer, Erik Schadde

**Affiliations:** 1 Institute of Physiology, University of Zurich, Zurich, Switzerland; 2 Institute of Anesthesiology, Institute of Anesthesiology, University of Zurich, University Hospital Zurich, Zurich, Switzerland; 3 Department of Pathology, Cantonal Hospital Winterthur, Zurich, Switzerland; 4 Division of Digestive Diseases, Rush University Medical Center, Chicago, Illinois USA; 5 Team Pathogenesis of viral hepatitis UMR INSERM 1052 - CNRS 5286 Centre de recherche en cancerologie de Lyon, Lyon, France; 6 Romain Parent Inserm U1052 Team #15 - Lyon Cancer Research Center, Lyon, France; 7 Department of Surgery, Division of Transplant Surgery, Rush University Medical Center, Chicago, Illinois USA; 8 Department of Surgery, Cantonal Hospital Winterthur, Zurich, Switzerland; 9 Department of Anesthesiology, University of Illinois at Chicago, Chicago, Illinois USA

**Keywords:** Mechanisms of disease, Hepatic stellate cells

## Abstract

Portal vein ligation (PVL) induces liver growth prior to resection. Associating liver partition and portal vein ligation (PVL plus transection=ALPPS) or the addition of the prolyl-hydroxylase inhibitor dimethyloxalylglycine (DMOG) to PVL both accelerate growth via stabilization of HIF-α subunits. This study aims at clarifying the crosstalk of hepatocytes (HC), hepatic stellate cells (HSC) and liver sinusoidal endothelial cells (LSEC) in accelerated liver growth. *In vivo*, liver volume, HC proliferation, vascular density and HSC activation were assessed in PVL, ALPPS, PVL+DMOG and DMOG alone. Proliferation of HC, HSC and LSEC was determined under DMOG *in vitro*. Conditioned media experiments of DMOG-exposed cells were performed. ALPPS and PVL+DMOG accelerated liver growth and HC proliferation in comparison to PVL. DMOG alone did not induce HC proliferation, but led to increased vascular density, which was also observed in ALPPS and PVL+DMOG. Activated HSC were detected in ALPPS, PVL+DMOG and DMOG, again not in PVL. *In vitro*, DMOG had no proliferative effect on HC, but conditioned supernatant of DMOG-treated HSC induced VEGF-dependent proliferation of LSEC. Transcriptome analysis confirmed activation of proangiogenic factors in hypoxic HSC. Hypoxia signaling in HSC induces VEGF-dependent angiogenesis. HSC play a crucial role in the cellular crosstalk of rapid liver regeneration.

## Introduction

The liver proliferates after rerouting portal vein blood flow without removal of liver mass, likely because portal vein blood contains trophic factors^1,2^ to maintain hepatocyte number and function^3^. Portal vein blood rerouting is used by surgeons to induce liver growth prior to liver resection in cases of small prospective (future) liver remnants (FLR) since 1986^4–6^. Portal vein branch embolization (PVE) of the hemi-liver with or without segment 4 is performed by interventional radiologists^7^ to increase volume by about 38% of the total liver volume within 4 to 6 weeks^6^. Portal vein ligation (PVL) by surgeons induces similar volume growth like PVE. However, volume increase with PVE or PVL is remarkably slow^8^. Recently, Associating Liver Partition and Portal vein ligation for Staged hepatectomy (ALPPS) was introduced as a surgical procedure in two stages, which combines PVL with parenchymal transection in a first stage, followed by hepatectomy in a second stage after only 7–10 days^9,10^. In animals models, ALPPS has been shown to increase portal venous blood flow per unit tissue just like PVL, but unlike PVL, the formation of portal collaterals over time is abrogated because of the parenchymal transection^11^. The consequence is persistent high portal flow in the small growing liver^12^. This high portal flow after ALPPS induces a persistent hypoxic environment with high levels of hypoxia-inducible factor 1α (HIF-1α) in the growing liver, whereas hypoxia does not persist in PVL^13^, likely due to the development of collaterals that decompress the portal hyperflow. It has been demonstrated that HIFα subunit stabilizers such as prolyl-hydroxylase inhibitors (PHI) like DMOG, which induce hypoxia signaling, also accelerate liver regeneration in rodents, and that factors increasing tissue oxygenation abrogate this effect^13,14^. It is also known that regeneration after partial resection is accelerated in mice genetically deficient for prolyl-hydroxylases^15,16^, which supports the role of hypoxia to accelerate liver regeneration.

Hypoxia signaling in the regenerating liver accelerates liver regeneration, but it is unclear which cell type (hepatocytes or non-parenchymal cells) transduces oxygen sensing into a proliferative signal in ALPPS. It is also not known what a short-term treatment of the normal liver with HIFα-stabilizing drugs does without portal vein rerouting. Current literature only provides information that genetic defects of von Hippel-Lindau syndrome and prolyl-hydroxylases - knockout mice with long term stabilization of HIFα subunits in the liver - lead to vascular malformations and liver steatosis^17^.

In this study, the effect of HIFα subunit stabilization using DMOG on the liver was investigated *in vivo* in rat models of PVL, ALPPS, PVL+ DMOG, DMOG-treatment of normal liver without portal vein rerouting. Volume changes and changes in histology were investigated. Additionally, isolated interrogation of the cellular components of the liver was performed using immortalized cell lines of hepatocytes (HC), hepatic stellate cells (HSC) and liver sinusoidal endothelial cells (LSEC) as well as primary cell isolates of HSC (pHSC) and LSEC (pLSEC) to better understand which cell types of the liver are involved in hypoxia sensing and how they affect acceleration of hepatocyte proliferation. Based on previous reports^18,19^, we hypothesized that HSC play a crucial role in hypoxia sensing and cellular crosstalk.

## Results

### Animal models

**Figure 1:**
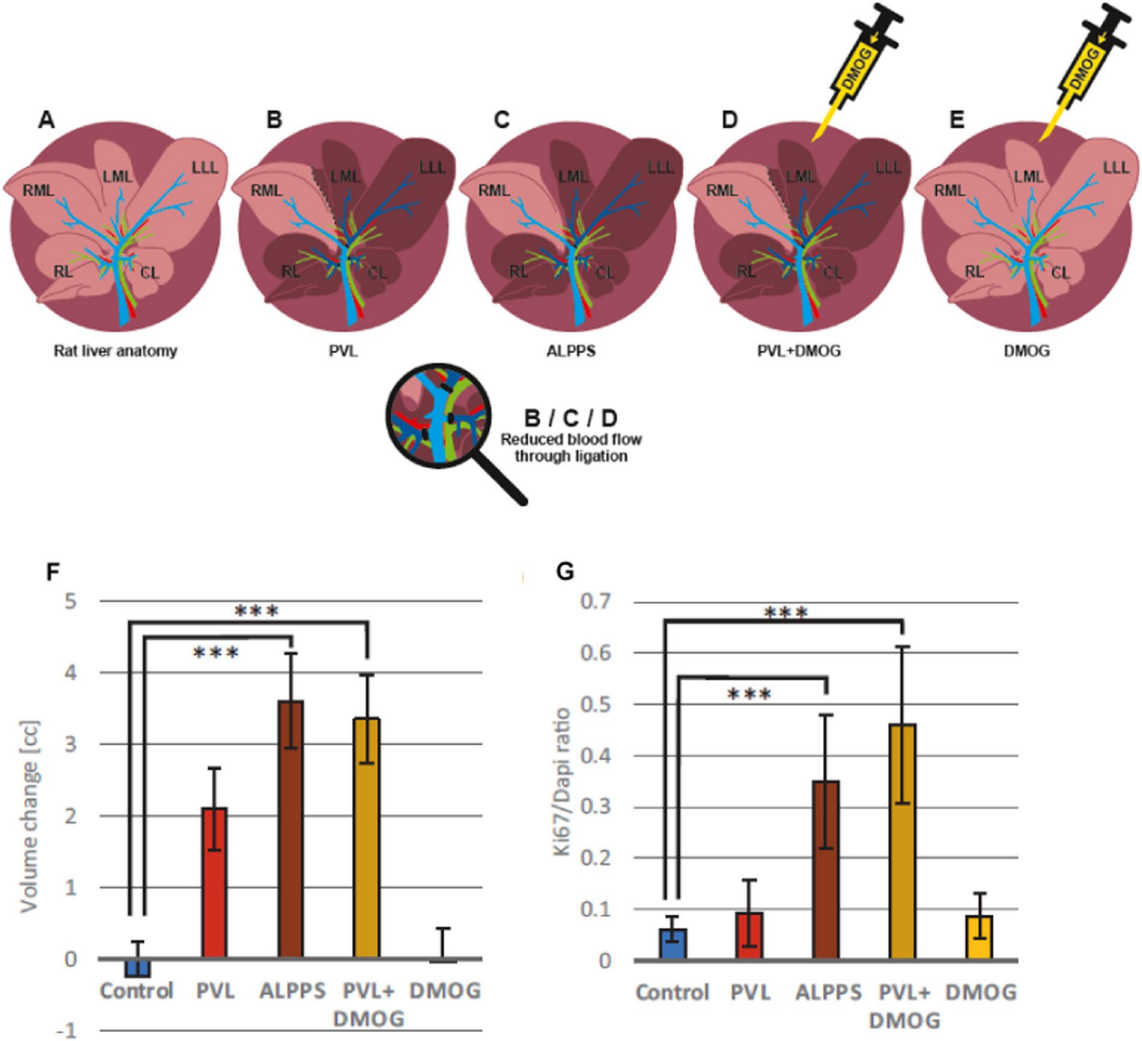
Rat models of regenerative liver surgery, volumetry and proliferation of rat livers after 72 h. **(A)** Rat liver anatomy with the right lobe (RL), the right and left middle lobe (RML, LML), the left lateral lobe (LLL), and the caudate lobe (CL). (**B**) Rat model of portal vein ligation (**PVL**): ligation of RL, LLL + LML, and CL. The dark liver area denotes the deportalized part of the rat liver with only arterial blood supply, the light browns one the liver with portal and arterial bi-perfusion. (**C**) Rat model of Associating Liver Partition and Portal vein ligation for Staged hepatectomy (**ALPPS**): transection of the ML along the ischemic line between RML and LML in addition to PVL. (**D**) Rat model of **PVL+DMOG**: i.p. administration of 200 µg/g body weight of dimethyloxalylglycine (DMOG) 12 h before PVL. (**E**) Rat model of **DMOG** application to liver: Repetitive (3x, up to 48 h) intraperitoneal (i.p.) application of DMOG without any rerouting of portal vein blood. (**F**) Growth assessment by volumetry using a small animal CT scanner after giving i.v. contrast to the animal directly after intervention and after 72 h to assess regeneration of the right middle lobe. Volume change is expressed as volume difference after 72 h in cubic centimeters after PVL, ALPPS, PVL+DMOG, DMOG alone and and normal liver control after intraperitoneal phosphate-buffered saline (control) application. (**G**) Proliferation assessment by histology. Ki-67 staining of the RML 72 h after PVL, ALPPS, PVL+DMOG, DMOG alone and normal liver control after intraperitoneal phosphate-buffered saline (control) application. n = 5 animals for controls and DMOG alone, n = 4 for ALPPS, PVL and PVL+DMOG. ***p < 0.001.

The right middle lobe (RML, 25% of the total liver volume) was used as FLR in models of PVL and ALPPS with 25% future liver remnant which have been established in this laboratory and were previously described Fig. 1A-C^13,20^. Control animals for PVL+DMOG, the prolyl-hydroxylase inhibitor dimethyloxalylglycine (DMOG) was injected intraperitoneally 12 h prior to PVL (Fig. 1D). For treatment of normal rat livers (Fig. 1E), DMOG was injected intraperitoneally and the injection was repeated after 24 and 48 h (phosphate-buffered saline, PBS, as control).

#### ALPPS and PVL+DMOG increase liver volume and proliferation

A total of n = 25 surgeries were performed in rats, n = 5 in each group (control, PVL, ALPPS, VPL+DMOG, DMOG alone). One animal died within 24 h after PVL, one animal died intraoperatively in the ALPPS group due to bleeding during hilar dissection and injury to the hepatic artery and one in the PVL+DMOG group within 24 h due to intraoperative hematoma resulting in a perioperative mortality rate of 12% (n = 3/25 for all animals). Portal re-routing with PVL induced a 2 cc increase in the RML volume by CT volumetry after 72 h. ALPPS as well as PVL+DMOG led to a 3.6 and 3.4 cc volume increase within 72 h. DMOG alone led to no increase in volume in the exemplarily selected RML of the rat liver (Fig. 1F). Hepatocyte proliferation in ALPPS and PVL+DMOG was demonstrated by an increased number of Ki-67 positive nuclei, while PVL and DMOG alone did not affect hepatocyte proliferation rate at 72 h (Fig. 1G).

#### Hypoxia and hypoxia signaling in liver cells in culture reduce cell proliferation

**Figure 2:**
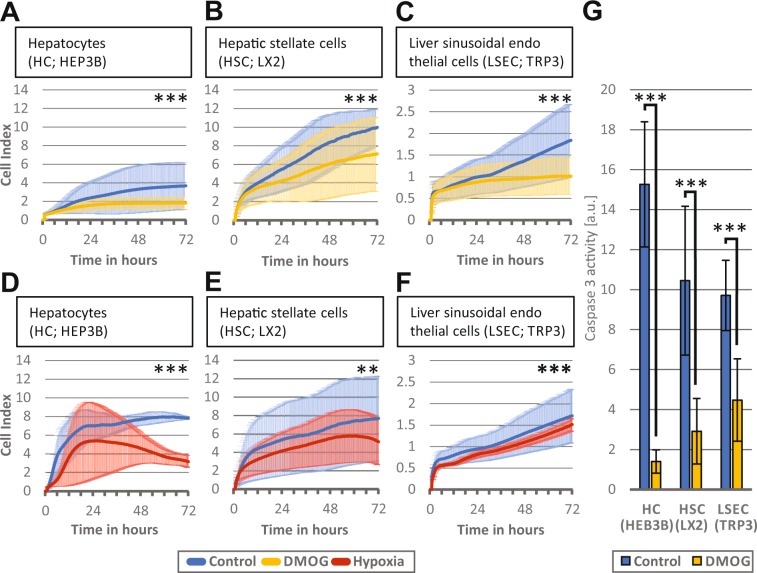
Effect of hypoxic signaling and hypoxia on liver cells *in vitro*. The iCELLigence system measures cell proliferation by impedance and thereby assesses proliferation of about 4500 plated cells over a time continuum. Bold lines represent mean values of the individual measurements, shadowed lines represent the SD of individual measurements. (**A**) Hepatocytes (HC) proliferation over 72 h after incubation with 1 mM dimethyloxalylglycine (DMOG) (**B**) Hepatic stellate cells (HSC) proliferation over 72 h after incubation with 1 mM DMOG and (**C**) Liver sinusoidal endothelial cells (LSEC) proliferation over 72 h after incubation with 1 mM DMOG. Control cells were exposed to phosphate-buffered saline instead of DMOG. (**D, E, F**) Proliferation of the 3 respective types were also cultivated in hypoxic conditions of 2% O, and proliferation was measured over 72 h. (**G**) HC, HSC, and LSEC were incubated with 1 mM DMOG for 72 h and caspase 3 activity was measured and normalized to the DNA content. **A-F**: n = 3 independent experiments (cell passages with two measurements) were performed for each group A-F and representative graphic outputs for each experiment are shown here: **p < 0.01; ***p < 0.001.

Incubation of HC, HSC and LSEC with DMOG *in vitro* reduced rather than enhanced proliferation of all three cell lines (n = 3 passages for each group, p < 0.001) (Fig. 2A-C). Similarly, exposure of HC, HSC and LSEC to hypoxic cell culture conditions resulted in a decreased cell proliferation (n = 3 passages for each group, p < 0.01) (Fig. 2D-F). To exclude toxicity of DMOG, HC, HSC and LSEC cells were incubated with 1 mM DMOG for 72 h and caspase 3 activity was measured as marker for apoptosis (Fig. 2G). In the presence of DMOG, caspase 3 activity was significantly reduced (n = 3 passages, p < 0.001), suggesting that increased apoptosis does not account for the decreased proliferation rate.

#### Conditioned media from hypoxic HSC induce LSEC proliferation

**Figure 3:**
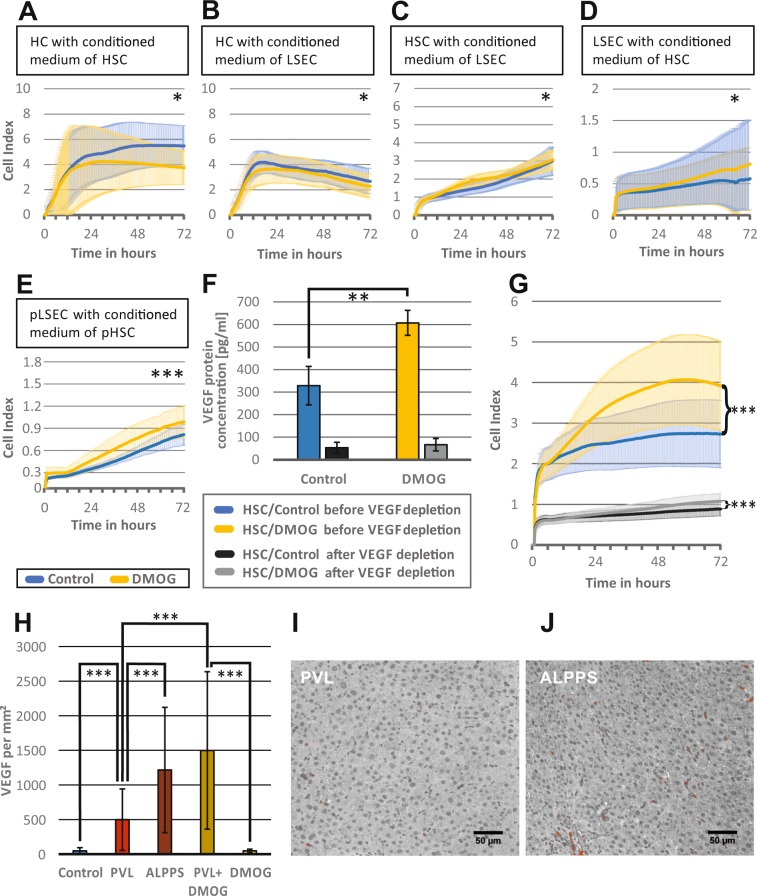
Effect of hypoxia-conditioned media on hepatocytes, hepatic stellate and endothelial cells *in vitro* and role of VEGF. The iCELLigence system measures cell proliferation by impedance and thereby assesses proliferation of about 4500 plated cells over a time continuum. Bold lines represent mean values of the individual measurements, shadowed lines represent the SD of individual measurements. (**A**) Hepatocyte (HC) proliferation over 72 h, after exposure to conditioned medium from hepatic stellate cells (HSC), incubated with 1 mM dimethyloxalylglycine (DMOG) for 24 h. (**B**) Hepatocyte (HC) proliferation over 72 h, after exposure to conditioned medium from Liver sinusoidal endothelial cells (LSEC) incubated with 1 mM DMOG for 24 h. (**C**) Hepatic stellate cell (HSC) proliferation over 72 h, after exposure to conditioned media from LSECs, incubated with 1 mM DMOG for 24 h. (**D**) LSEC proliferation over 72 h after exposure to conditioned medium from HSC, incubated with 1 mM DMOG for 24 h. (**E**) Validation of the finding that DMOG-exposed HSC supernatants stimulate LSEC proliferation, using primary cell culture of HSC (pHSC) and LSEC (pLSEC). pLSEC proliferation over 72 h after exposure to conditioned medium from pHSC, incubated with 1 mM DMOG for 24 h. (**F**) VEGF concentration as measured by enzyme-linked immunosorbent assay (ELISA) in supernatants of HSC cultured without (control = blue bar) and incubated with 1 mM dimethyloxalylglycine (DMOG = yellow bar) for 24 h, followed by vascular endothelial growth factor (VEGF) depletion (black and grey bars, respectively). (**G**) Liver sinusoidal endothelial cells (LSEC) proliferation measured for 72 h, after exposure to control medium (blue) and DMOG-conditioned medium (yellow) from HSC before VEGF depletion, and after VEGF depletion (control = black and DMOG = yellow), respectively. **A-C, F-G**: n = 3 independent experiments with two measurements for each passage), **D-E:** n = 4 independent experiments with two measurements for each passage were performed for each group. (**H**) Histological quantification of VEGF, in immunohistochemistry staining. Representative regions of interest (ROI) of the right middle lobe (RLM), measured 72 h after PVL, ALPPS, PVL+DMOG, DMOG and controls were evaluated. VEGF-positive areas were marked red using the threshold function of Image J, counted and expressed as counts per mm^2^. (**I**, **J**) Representative photomicrograph of immunohistochemistry with VEGF (red) of rat liver RML 72 h after PVL or ALPPS. n = 5 animals for controls and DMOG alone, n = 4 for ALPPS, PVL and PVL+DMOG. 10 ROIs per animal were counted. *p < 0.05; **p < 0.01; ***p < 0.001.

Since there was no pro-proliferative effect of hypoxia or DMOG on HC and non-parenchymal cells in cell culture, cellular crosstalk was examined using conditioned media. In a first approach, HC were incubated with either medium from DMOG-treated HSC or LSEC. As shown in Fig. 3A,B, the conditioned media of DMOG-treated HSC and LSEC impaired growth of HC (n = 3 passages for each group, p < 0.05). Then, HSC were incubated with medium from DMOG-treated LSEC. There was an increase of HSC proliferation with medium from DMOG-exposed LSEC (n = 3 passages, p < 0.05) (Fig. 3C). Also, medium from DMOG-treated HCS significantly accelerated proliferation of LSEC (n = 3 passages, p < 0.05) (Fig. 3D).

### Confirmation of acceleration of LSEC proliferation by conditioned media form DMOG-exposed HSC in primary cell culture (pLSEC, pHSC)

Immortalized cells lines may react differently from cells after primary isolation from the liver. The findings of screening for the effect of conditioned media of DMOG-treated HSC on LSEC proliferation was therefore validated *in vitro* in pHSC and the pLSEC. Results are shown in Fig. 3E, where similarly to Fig. 3D DMOG-treated pHSC supernatants accelerated proliferation of pLSEC (n = 3 passages, p < 0.001).

#### Transcriptome analysis of DMOG-treated HSC and conditioned LSEC revealed increased angiogenesis pathways

For a more detailed understanding of the interaction and the signaling between HSC and LSEC, transcriptome analysis was performed of DMOG-treated HSC. Furthermore, LSEC, incubated with the conditioned media of DMOG-treated HSC, were analyzed. The transcriptome data from this study are uploaded in the GEO data repository under the following accession number: Series record GSE131168. In DMOG-treated HSC, 2261 genes were differentially expressed, 759 transcripts were up-regulated and 1502 down-regulated with a fold change of 2 or more. Functional annotation of the transcripts was performed using DAVID^21,22^. The cluster ‘HIF-1 pathway’ referred to 9 up-regulated and 3 down-regulated genes, the cluster ‘angiogenesis’ to 7 up-regulated and 12 down-regulated genes, the cluster vascular growth factor ‘VEGF pathway’ to 4 up-regulated and 2 down-regulated genes, the cluster ‘apoptosis’ to 33 up-regulated and 40 down-regulated genes, and the cluster ‘p53 pathway’ revealed 6 up-regulated and 4 down-regulated genes (Supplementary Table 1). In LSEC, incubated with conditioned medium from DMOG-treated HSC, 270 genes were differentially expressed, of which 129 were up- and 141 down-regulated. The ‘HIF-1 pathway’ cluster referred here to 6 up-regulated genes, the cluster ‘angiogenesis’ also to 6 upregulated genes, 24 up-regulated and 18 down-regulated genes were functionally linked to apoptosis (Supplementary Table 2).

#### Hypoxic HSC produce VEGF and stimulate LSEC proliferation

The finding that VEGF and angiogenesis pathways were strongly represented at the transcriptome level led to the measurement of VEGF in conditioned media of DMOG-treated HSC. VEGF concentration doubled in conditioned medium of DMOG-treated HSC (329 pg/ml *vs* 607 pg/ml, p < 0.01) (Fig. 3F).

To evaluate the relevance of VEGF to induce LSEC proliferation, VEGF protein depletion was performed in medium from DMOG-treated or control HSC, which was confirmed by ELISA (Fig. 3F). When exposing HSC with VEGF-depleted medium from DMOG-exposed HSC, proliferation was strongly reduced, but LSEC still proliferated significantly more in media of DMOG-treated HSC than in media of non-treated HSC (p < 0.001), suggesting that other (angiogenetic) factors are present in the supernatant of DMOG treated HSC. The much lower proliferation level after VEGF depletion however shows that VEGF is the main growth factor to support proliferation of LSEC (Fig. 3G).

To verify this higher VEGF presence *in vivo* during rapid liver regeneration, we performed immunohistochemistry for VEGF following slow regeneration after 72 h PVL with moderate hypoxia, or rapid regeneration after ALPPS with pronounced hypoxia, as well as after PVL+DMOG and DMOG alone. Though there was no significant difference between DMOG application and control (47.7 *vs* 48.3 counts per mm^2^, p = 0.96), VEGF was moderately increased 72 h after PVL (500 counts per mm^2^, p < 0.0001 *vs* control) and highly present after the rapid regeneration models ALPPS (1216 counts per mm^2^, p < 0.0001 *vs* control and p = 0.0002 *vs* PVL) and PVL+DMOG (1498 counts per mm^2^, p < 0.0001 *vs* control and *vs* PVL; Fig. 3H). According VGEV staining in PVL and ALPPS is shown in Fig. 3I,J, respectively.

#### Liver tissue undergoing rapid hypertrophy shows hypervascularity by vWF and CD34 staining

**Figure 4:**
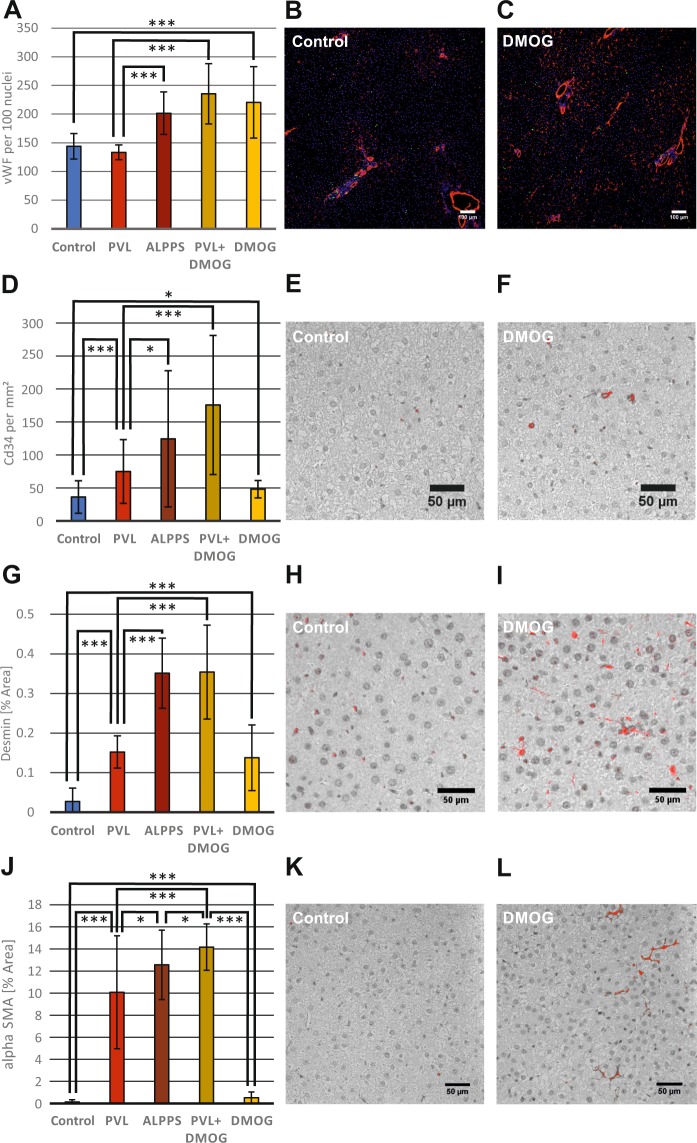
Angiogenesis in rapid liver regeneration models and the activation of hepatic stellate cells *in vivo*. (**A**) Histological quantification of the endothelial marker von Willebrand factor (vWF) after immunofluorescence staining. Regions of interest (ROI) of the right middle lobe (RML) were examined in rat models of PVL, ALPPS, PVL+DMOG, DMOG and control. Microvascular cross sections with a diameter below 20 µm were counted and related to DAPI-positive nuclei. (**B**,**C**) Representative photomicrograph of immunofluorescence staining for vWF (red), the proliferation marker Ki67 (green) and DAPI nuclear staining (blue) of rat liver RML 72 h after intraperitoneal phosphate-buffered saline (control) or DMOG administration. (**D**) Histological quantification of CD34, which is expressed in endothelial cells only during their differentiation, in immunohistochemistry staining. ROI of the right middle lobe (RLM) were evaluated 72 h after PVL, ALPPS, PVL+DMOG, DMOG and controls. CD34-positive areas were marked red using the threshold function of Image J, counted and expressed as counts per mm^2^. (**E**,**F**) Representative photomicrograph of immunohistochemistry with CD34 of rat liver RML 72 h after DMOG administration or control. (**G**) Histological quantification of desmin, a marker for activated HSC, in immunohistochemistry staining. ROI of the right middle lobe (RLM) were evaluated 72 h after PVL, ALPPS, PVL+DMOG, DMOG and controls. The desmin-stained area was marked red using the threshold function of Image J, measured and expressed as percent of the total area. (**H**,**I**) Representative photomicrograph of immunohistochemistry with desmin of rat liver RML 72 h after DMOG administration or control. (**J**) Histological quantification of α-smooth-muscle actin (αSMA), a histological marker for stellate cells in immunohistochemistry staining. ROI of the right middle lobe (RLM) were evaluated 72 h after PVL, ALPPS, PVL+DMOG, DMOG and controls. The area stained for αSMA was marked red using the threshold function of Image J, measured and expressed as percent of the total area. (**K**,**L**) Representative photomicrograph of immunohistochemistry with αSMA of rat liver RML 72 h after DMOG administration or control. n = 5 animals for controls and DMOG alone, n = 4 for ALPPS, PVL and PVL+DMOG. 10 ROIs per animal were counted. *p < 0.05; ***p < 0.001.

To better understand a putative effect of VEGF produced by HSC with activated hypoxia signaling during rapid liver regeneration, we performed immunohistochemistry for the endothelial marker von Willebrand factor (vWF) in rat livers to detect differences in endothelial density *in vivo*, following slow regeneration after 72 h PVL with moderate hypoxia, or rapid regeneration after ALPPS with pronounced hypoxia. Quantification showed that vascular density did not change after 72 h in slow regeneration (PVL), but significantly increased in rapid regeneration after ALPPS as well as after PVL+DMOG (p < 0.001 PVL *vs* ALPPS or PVL+DMOG) (Fig. 4A). Vascular density after DMOG application to normal livers (Fig. 4B) with PBS; Fig. 4C with DMOG) was comparable to ALPPS and PVL+DMOG (Fig. 4A) using vWF staining. Of note, no volume increase or increased Ki-67 staining of HC were observed in these livers (see Fig. 1F,G).

Like vWF, CD34 is a common LSEC/endothelial cell marker in the liver during endothelial differentiation and was used to additionally evaluate vascularity. Both vWF and CD34 are also expressed in TRP3 cells, which were used for cell culture experiments in this study. CD34 staining demonstrated a significant increase in staining in ALPPS and PVL+ DMOG compared to PVL (p < 0.05 PVL *vs* ALPPS or p < 0.001 PVL *vs* PVL+DMOG) (Fig. 4D). Also, livers after PVL had increased staining compared to livers from control animals (Fig. 4E) or livers after the injection of DMOG alone (Fig. 4F).

### Activation of HSC *in vivo* in ALPPS, DMOG+PVL and DMOG treated livers

Because conditioned media from hypoxic HSC induced LSEC proliferation in cell culture, and because DMOG-treated HSC were found to differentially express over 2000 genes compared to non-hypoxic HSC, HSC were interrogated for activation *in vivo* using desmin staining. Increased desmin synthesis and formation of desmin-containing intermediate filaments (IFs) are signs of transdifferentiation of HSC into myofibroblast-like cells. These desmin-enriched myofibroblast-like cells are the source of fibrotic extracellular matrix in chronically diseased liver^23^. We found that at 72 h after the procedures desmin was increased in the growing livers in ALPPS and PVL+DMOG compared to control livers (desmin signal 0.35% and 0.35% *vs* 0.03% total area, p < 0.001) (Fig. 4G). DMOG application in normal livers also activated HSC (0.14% desmin area *vs* 0.03%, p < 0.001) (Fig. 4H with PBS; Fig. 4I with DMOG).

We also used a second activation marker for HSC, α-smooth muscle actin (αSMA). The findings showed a correlation to the results of the desmin staining with an increase in ALPPS (αSMA signal 12.6% *vs* 0.13% total area, p < 0.001) and PVL+DMOG (14.2% *vs* 0.13% total area, p < 0.001) livers more than in PVL livers (10.1% *vs* 0.13% total area, p < 0.001) (Fig. 4J). Even DMOG application in normal livers activated HSC (0.52% *vs* 0.13% total area, p = 0.0004) (Fig. 4K with PBS; Fig. 4L with DMOG).

### Possible mechanism for rapid liver regeneration

**Figure 5:**
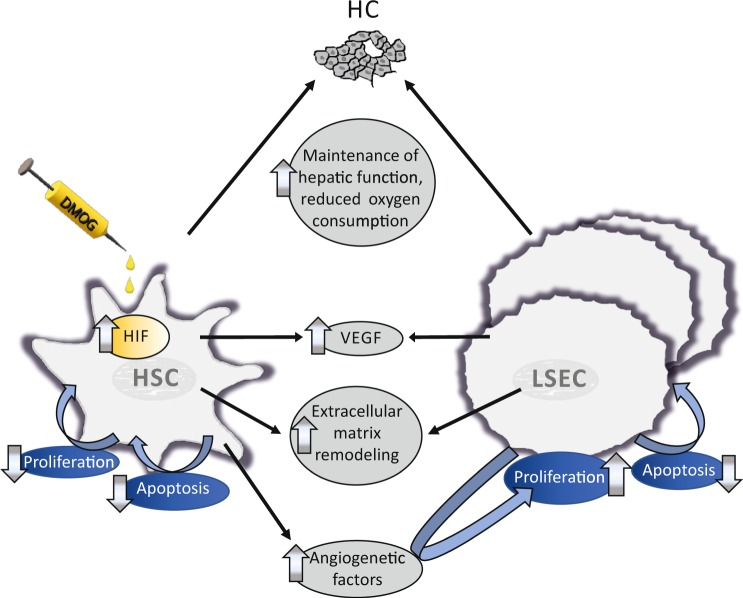
Transcriptome analysis of hypoxic stellate cells and liver sinusoidal endothelial cells, exposed to the conditioned medium from hypoxic stellate cells. Hepatic stellate cells (HSC) were incubated with 1 mM dimethyloxalylglycine (DMOG) for 24 h, supernatants were given as conditioned media to liver sinusoidal endothelial cells (LSEC) for 24 h and RNA extracted. Transcriptome analysis, functional annotation of differently regulated transcripts using the Database for Annotation, Visualization, and Integrated Discovery (DAVID), and evaluation of these genes using the National Center for Biotechnology Information (NCBI) gene database revealed a complex, multifunctional induction of angiogenesis and extracellular matrix remodeling through DMOG-treated HSC and self-enforcing, pro-proliferative changes in the transcriptome of LSEC incubated with conditioned medium of DMOG-treated HSC. Hypoxia-inducible factor (HIF) pathways and particularly vascular endothelial growth factor (VEGF) pathway were upregulated. Full transcriptome data sets have been uploaded to the NCBI GEO data repository.

Genes referred to in the clusters ‘HIF-1 pathway’, ‘VEGF pathway’, ‘angiogenesis’, ‘apoptosis’, ‘p53 pathway’, ‘ECM-ECM-receptor-interaction’ by DAVID (Supplementary Tables 1 and 2) were further evaluated using the National Center for Biotechnology Information (NCBI) gene database. Dimethyloxalylglycine treatment of HSC and incubation of LSEC with conditioned media from HSC after a 24-hour DMOG exposure caused a complex change in the transcriptome of both HSC and LSEC that led to induction of LSEC proliferation through influencing multiple pathways. These findings are summarized in Fig. 5.

## Discussion

This study demonstrates that hypoxia signaling in HSC leads to a VEGF-driven induction of proliferation of LSEC in *vitro*. Also, *in vivo* data from the rapid liver regeneration models ALPPS in rats reveal that rapid regeneration is associated with an increase in vWF and CD34-positive endothelium in ALPPS compared to PVL and activation of HSC as shown by desmin staining and αSMA staining. All of these effects can also be induced by treating the animals with DMOG prior to PVL.

DMOG’s primary known function is to stabilize the HIF𝛼 protein subunits^24^. Normal livers without portal vein rerouting demonstrate changes in the density of their vascularity by vWF staining after treatment with DMOG without proliferation of HC, assuming that the increased vascularity observed after ALPPS and PVL+DMOG is probably not secondary to HC proliferation, but an independent process. The cell culture data presented here suggest that this process may be initiated by HSC being the DMOG/hypoxia-sensing entity. No other pro-proliferative effect of hypoxia-conditioned media of the liver constituents HC, HSC or LSEC on each other could be detected in these experiments. Pathway analysis of the RNA expression of the HIF𝛼-stabilized HSC and LSEC, stimulated by supernatant from hypoxic HSC, suggests a largely VEGF-dependent angiogenesis, metabolic reprogramming and extracellular matrix remodeling (Fig. 5). Vascular endothelial growth factor concentration doubled in the supernatant of HIF𝛼-stabilized HSC, and VEGF depletion experiments provided causal evidence that the proliferative effect of HSC supernatants on LSEC is mainly dependent on VEGF.

The crosstalk between HSC and LSEC has been investigated mostly in the context of liver fibrosis and cirrhosis^25^, because activation of HSC and angiogenesis are mainly associated with liver fibrosis. Under physiological conditions, HSC are known to support the LSEC and their phenotype through the release of VEGF^26^. While VEGF may be constitutively expressed in HSC under normoxic conditions, our cell culture data showed that under hypoxic conditions, VEGF production by HSC is increased. We propose that HSC function as hypoxia sensors in the liver, and trigger angiogenesis not only in fibrosis and cirrhosis, but also in rapid liver regeneration. Interestingly, this effect can be induced by HIF𝛼−stabilizing drugs, while angiogenesis triggered by hypoxia signaling (induced with DMOG) occurs without proliferation of hepatocytes *in vivo*. Vascular endothelial growth factor plays a central role in this signaling cascade, although VEGF depletion from the conditioned medium of HSC did not entirely abolish the stimulating effect of DMOG-conditioned HSC media. Other pro-angiogenic factors such as endothelin 1 (EDN1)^27^, and a decrease of anti-angiogenetic factors by HSC could occur and will have to be examined in the future.

Slow liver regeneration after PVL and PVE may simply be due to a higher concentration of essential and non-essential trophic factors^28^ for HC besides initial tissue hypoxia. In contrast, proliferation after ALPPS may be accelerated, due to the persistently high level of hypoxia in the liver, which induces additional angiogenesis, mediated by activated HSC. The tissue hypoxia in the growing liver is either the result of the arterial buffer response due to the portal hyperflow induced in liver rerouting maneuvers and partial hepatectomy^29–31^ or the result of a relative hypermetabolism of the hyperperfused and regenerating liver^32^. In PVL or PVE, portal hyperflow is a short-lived phenomenon of only hours, because it is counteracted by portal vein shunting across sinusoids with large collaterals developing over several days^11^. In ALPPS, hypoxia and thereby HSC activation continues and only ceases after the expansion of the sinusoidal capillary bed and the increase in liver size. Any process that inhibits collateral formation in portal vein rerouting – like ALPPS and many of its modifications which render parenchyma between lobes not viable for collateralization - leads to prolonged and sustained regeneration^11^ until the FLR reaches full liver size, and hypoxic signaling in HSC discontinues. The decrease in hypoxia signaling may be a stop-signal of hepatocyte proliferation^33^ after normalization of portal flow due to expansion of the liver sinusoidal endothelial bed to full size. It has been hypothesized that hypoxia has direct pro-proliferative effect on HC^15,16,34,35^, but we now postulate that HC are only effector cells of a process that is initiated and maintained by HSC and may be mediated by LSEC proliferation.

The cross talk between LSEC proliferation and HC has to be explored in detail. Angiogenesis may lead, after the first three days, to a better vascular perfusion capacity, i.e. the distribution with nutrients and hepatotropic substances from the intestine and improve liver function by shorter diffusion of metabolic substance exchange and removal^36^. However, acceleration of HC proliferation in ALPPS and PH is observed already after 24 h^13,37^ which may be too early to be explained by angiogenesis. It is more likely that proliferating liver endothelial cells and LSEC have an angiocrine role and stimulate HC proliferation through the VEGF pathway and Id1-dependent secretion of paracrine trophogens like hepatic growth factor (HGF) and Wnt as previously described^36^.

A limitation of this study is that there are many endothelial markers for liver endothelial cells and LSEC. We picked vWF and CD34 due to the presence of both markers on the immortalized cell cultures we used as screening tools. A further evaluation of vascular markers development across the liver architecture is needed to understand how endothelial growth accelerates liver regeneration in ALPPS and PLV+DMOG. Second, functional proof of the role of HSC for rapid liver regeneration in models of stellate cell depletion or silencing and animals genetically deficient for HIF𝛼 and VEGF is still pending and underway.

Prolyl hydroxylase domain-containing enzymes degrade HIF𝛼 subunits and can be blocked by PHI such as DMOG^38^. Prolylhydroxylase inhibitors are interesting novel drugs that simulate hypoxic signaling in all cells of the body. The first-in-class PHI Roxadustat met all primary efficacy endpoints in three clinical phase III trials for treatment of renal anemia^39^. This study suggests they PHI may also be potent inducers of angiogenesis in the liver, independently of hepatocyte division. While PHI do not stimulate tumor growth in animals^16,24^, they may convey a cytoprotective effect to hepatocytes as shown by the reduction in apoptosis in this cell culture model. The clinical effects of liver ischemic preconditioning have been attributed to the HIF𝛼 pathway^40^. Roxadustat has recently been approved for human use in China^41^. In a clinical setting, PHIs could offer accelerated liver regeneration in partial hepatectomy, portal vein rerouting and after toxic or ischemic insults.

In conclusion, there is strong cell-culture and *in-vivo* evidence that hypoxia signaling by HSC with resulting angiogenesis plays a role in the acceleration liver regeneration seen in ALPPS and PVL+DMOG. This will have to be further evaluated in animals with HSC depletion or lack of hypoxia signaling in HSC.

## Methods

*Animals, experimental approach and volumetry* Approval for the animal experiments with male Wistar rats (Charles River, Sulzfeld, Germany) was obtained from the Veterinary Office of the Canton of Zurich, Switzerland (number ZH60/2014). Female animals were not considered because of the hormone cycle, which could impact on organ regeneration. Experiments were performed in compliance with the guidelines for animal experiments by the Swiss Academy of Medical Sciences and the Federation of European Laboratory Animal Science Associations. Food and water were provided ad libitum. Animals were kept at 12/12-hour light/dark cycle in ambient temperature of 22 ± 2 °C. A sample size calculation was not performed. The group size is based on former experiences, which allows a proper statistical analysis with the least number of animals possible respecting 3 R. Experiments were started (randomization procedure) after seven days of accommodation in ventilated cages (n = 5 rats for each group: control, PVL, ALPPS, PVL+DMOG, DMOG alone). Rat models of PVL and ALPPS have been established previously^37,42–44^. The RML was used as the FLR, and PVL as well as ALPPS were performed according to previous experiments^13,20^. For PVL+DMOG, 200 µg/g body weight of the PHI DMOG (Frontier Scientific, Newark, DE, USA), was injected intraperitoneally 12 h prior to PVL. In the DMOG group, the 200 µg/g body weight of DMOG was injected intraperitoneally after sham surgery and the injection was repeated transcutaneously after 24 and 48 h (with PBS as control). Before and 72 h after the respective procedures, animals underwent small animal computer tomography (CT) volumetry as previously described^13^ to assess change of volume change of the FLR. The animals were sacrificed at 72 h for tissue procurement.

### Immunofluorescence for Ki67, DAPI and vWF

Cryosections of 6 µm were fixed using 4% paraformaldehyde (Sigma Aldrich/Merck, Darmstadt, Germany). After 10 min at room temperature, slides were kept in ice-cold PBS, washed and blocked at room temperature for 1 h using PBS +2% bovine serum albumin (BSA) (KPL, Gaithersburg, MD, USA) + 0.3% Triton X-100 (Sigma Aldrich/Merck). Probes were incubated overnight at 4 °C with the following primary antibodies: rabbit anti-Ki67 (Abcam, Cambridge, UK) diluted 1:400, mouse anti-vWF (Santa Cruz, Dallas, TX, USA) diluted 1:100 in blocking buffer. After washing, staining was performed with secondary antibodies goat anti-mouse Alexa 488 (Life Technologies, Zug, Switzerland), goat anti-rabbit Alexa 568 (Abcam), 4,6-diamidin-2-phenylindol (DAPI; Roche, Rotkreuz, Zug, Switzerland) in a 1:1000 dilution in PBS for 1 h at room temperature. Slides were mounted with ProLong Gold antifade reagent (Life Technologies).

Images were acquired with either the widefield microscope Leica DMI 6000 B, the fluorescence monochrome camera Leica DFC 9000 GTC or the color imaging camera Leica DFC 420 C. Leica Application Suite X 3.0.2.16120 (Leica Microsystems, Heerbrugg, Switzerland) or the slidescanner Axio Scan.Z1, using ZEN blue image processing and analyzing software (Zeiss, Feldbach, Switzerland). Automated counting of DAPI- and Ki67-positiv nuclei was performed with ImageJ 1.51 k (Wayne Rasband, National Institutes of Health, USA), using the watershed tool and the Triangle dark auto-threshold for Ki67, an infinity of 2 µm and a circularity of 0.5–1.0. Vascular density was assessed in an area of 0.211 mm^2^ (500 × 1000 Pi) per ROI, excluding artefacts and vessels with a diameter of more than 30 µm.

### Immunohistochemistry for VEGF, CD34, desmin and αSMA

Formalin fixed tissue was embedded in paraffine and sections of 1.5 µm were prepared. The fully automated Leica Bond III stainer (Leica Biosystems, Nussloch, Germany) was used to incubate slides in Bond Epitope Retrieval Solution 2 (Biosystems, Muttenz, Switzerland) for 20 min at 95 °C and then at room temperature for 15 min with 1:100 diluted rabbit anti-VEGF (Abcam, Cambridge, UK), with 1:50 diluted rabbit anti-CD34 (Abcam, Cambridge, UK), with diluted 1:50 mouse anti-desmin (CellMarque, Rocklin, CA, USA) or with 1:400 diluted rabbit anti-αSMA (Abcam, Cambridge, UK) antibodies. Visualization was achieved using the Leica Bond Polymer Refine Detection technique (Leica Biosystems, Nussloch, Germany) according to the manufacturer’s instructions. For VGEF, CD34, and αSMA quantification in immunohistochemistry, a 20x objective with aperture was used and the stained area as a fraction of a total area of 0.245 mm^2^ (864 × 648 Pi) was measured with ImageJ 1.51 k.

*Cell culture, experimental approach, cell proliferation* Hep3B human hepatoma cells, kindly provided by Bruno Stieger, University Hospital Zurich, were cultured in DMEM with high glucose (Dulbecco’s Modified Eagle Medium; Life Technologies) supplemented with 110 mg/L sodium pyruvate, 10% fetal bovine serum (FBS; Life Technologies) and penicillin (100 U/mL)/streptomycin (100 μg/mL) (Life Technologies). LX-2 human hepatic stellate cells, kindly provided by Scott Friedman, Mount Sinai School of Medicine, New York, NY, USA, were grown as described above, but without pyruvate. TRP3 human liver sinusoidal endothelial cells, kindly provided by Birke Bartosch, INSERM, Centre de Recherche en Cancérologie de Lyon, France, were cultured in 0.1% porcine gelatin-coated plates in MCDB131 medium (Life Technologies) with 10% Fetal Clone II Serum (GE Healthcare Lifesciences, South Logan, UT, USA), 10 mmol/l GlutaMAX (Thermo Fischer Scientific, Waltham, MA, USA), 1 µg/ml hydrocortisone (Sigma Aldrich/Merck), penicillin/streptomycin as described, 250 µg/ml adenosine 3′, 5′-cyclic monophosphate (Sigma Aldrich/Merck) and 50 µg/ml endothelial cell growth supplement from bovine tissue (Sigma Aldrich/Merck).

Each cell line was incubated with medium containing 1 mM DMOG (final concentration) for 72 h (according control group with medium only) and proliferation was determined. For crosstalk experiments, a specific cell type was exposed to DMOG for 24 h. This medium (‘conditioned medium’) was then transferred to another cell line and incubated for 72 h (medium from control group).

For hypoxia experiments cells were exposed to 2% oxygen and 5% carbon dioxide in a humified surrounding in a regular incubator (INCO 2 153, Memmert, Schwabach, Germany) while control cells were kept at control conditions (21% oxygen, 5% carbon dioxide, humified surrounding) in an identical incubator.

Cell proliferation was measured for 72 h using the impedance-based RTCA iCELLigence system (ACEA Biosciences, Inc, San Diego, CA, USA). Installation and calibration was carried out according to the manufacturer’s instructions. 4500 cells per well, or 2000 cells per well for experiments with conditioned media, were seeded into an E-Plate L8. Cells were allowed to adhere for 30 min (HSC, HC) or 120 min (LSEC) before starting the measurement. Impedance was measured every 15 min over 72 h. Cell confluency was checked microscopically at the end to exclude over-confluent plates. Data were analyzed with the RTCA Data Analysis Software 1.0.

### Primary cell culture (pHSC and pLSEC) and experimental setup pLSEC with conditioned medium of pHSC

To verify LSEC proliferation in conditioned medium from HSC primary cell culture experiments were performed. A human primary liver sinusoidal endothelial cell line as well as a human primary liver stellate cell line were purchased from Innoprot, Derio-Bizkaia, Spain (P10652 and P10653, respectively). 6 well plates were coated with poly-l-lysine (Innoprot) according to the manufacture’s protocol at 37 °C overnight. One hundred thousand pHSC were then added to each well in culture medium (Innoprot; medium: stellate cell basal medium, fetal bovine serum, stellate cell growth supplement, penicillin/streptomycin solution). After reaching 90% confluency pHSC were incubated with PBS as a control and DMOG (1 mM) for 24 h as previously described for HSC. After 24 h the supernatants were transferred to pLSEC to quantify proliferation.

iCELLigence system plates were coated with fibronectin (Innoprot) according to the manufacturers protocol for overnight at 37 °C, and pLSEC were seeded at a density of 12000/ml and incubated with the collected supernatants of the pHSC.

*VEGF ELISA, VEGF depletion experiments* Vascular endothelial growth factor was measured using enzyme-linked immunosorbent assay (ELISA) according to manufacturer’s instructions (R&D Systems, Abingdon, UK). For VEGF depletion, 24-well plates were coated overnight with a capture antibody from the ELISA kit in a 1:120 dilution in PBS, followed by two washing steps. 150 µl conditioned medium of DMOG-treated or untreated HSC was added to the VEGF antibody-coated well for 1 h at room temperature under constant movement. This step was repeated once. Subsequently, LSEC were then incubated with the VEGF-depleted conditioned medium from HSC, and proliferation was measured for 72 h, as described.

*Transcriptome and GeneChip microarray assay* Total RNA was extracted from cultured LX-2 and TRP3 according to the manufacturer’s instructions using the RNeasy Mini Kit (Qiagen, Hilden, Germany) after 24 h of incubation with DMOG or supernatants of DMOG-treated HSC. Sample preparation for microarray hybridization was performed using the Affymetrix GeneChip WT PLUS Reagent Kit according to the Manifacturers Instructions (Affymetrix, Inc., Santa Clara, CA, USA). Sample processing was performed at an Affymetrix Service Provider and Core Facility, “KFB - Center of Excellence for Fluorescent Bioanalytics” (Regensburg, Germany; www.kfb-regensburg.de). Summarized probe set signals in log2 scale were calculated by using the GCCN-SST-RMA algorithm with the Affymetrix GeneChip Expression Console v1.4 Software. After exporting into Microsoft Excel, average signal values, comparison fold changes and significance P values were calculated. Probes with an at least 2-fold change and a p value lower than 0.01 were considered significantly regulated. Functional annotation of differently regulated transcripts was performed using the Database for Annotation, Visualization, and Integrated Discovery (DAVID)^21,22^ and genes referred to in the clusters ‘HIF-1 pathway’, ‘VEGF pathway’, ‘angiogenesis’, ‘apoptosis’, ‘p53 pathway’, ‘extracellular matrix (ECM)-ECM-receptor-interaction’ were further evaluated using the National Center for Biotechnology Information gene database (NCBI, U.S. National Library of Medicine, Bethesda, MD, USA).

*Statistical analysis* Unpaired Student *t* test (GraphPad, San Diego, CA, USA) was used to compare the animal models after confirming normal distribution with the Kolmogorov-Smirnov test. Due to the large number of cells assessed in the cell proliferation assays, the *in vitro* studies consisted of a minimum of three independent experiments (passages of cells from a frozen state) performed in duplicate measurements. The exact number of passages used for each experiment is given in the results section. For statistical evaluation of cell growth, a linear regression including a bias corrected and accelerated bootstrapping with 1000 random samples and a confidence interval of 95% was performed. Requirements were controlled by a residual analysis. Unpaired Student *t* test was used to compare the ELISA values between groups.

## Supplementary information

Supplementary Dataset.

## Data Availability

Data repository for transcriptome data NCBI’s GEO, URL: https://www.ncbi.nlm.nih.gov/geo/query/acc.cgi?acc=GSE131168
