# Supplementary material for: Hypoxia sensing by hepatic stellate cells leads to VEGF-dependent angiogenesis and may contribute to accelerated liver regeneration
Source: Sci Rep. 2020 Mar 9;10:4392. doi: 10.1038/s41598-020-60709-9 (PMC7062856; doi:10.1038/s41598-020-60709-9)
Supplement: Supplementary file 1 — Supplementary Dataset. [file 41598_2020_60709_MOESM1_ESM.docx]

**Supplementary information**

Hypoxia sensing by hepatic stellate cells leads to VEGF-dependent angiogenesis and may contribute to accelerated liver regeneration

**Short title:** Hypoxic stellate cells accelerate liver regeneration

Konstantin Dirscherl,^1,2^ Martin Schläpfer,^1,2^ Birgit Roth Z’graggen,^1,2^ Roland H Wenger,^1^ Christa Booy,^1,2^ Renata Flury-Frei,^3^ Rita Fatzer,^3^ Costica Aloman,^4^ Birke Bartosch,^5^ Romain Parent,^6^ Vartan Kurtcuoglu,^1^ Diane de Zélicourt,^1^ Donat R. Spahn,^2^ Beatrice Beck Schimmer,^1,2,9^ Erik Schadde,^1,7,8,^ **^*^**

^1^ Institute of Physiology, University of Zurich, Zurich, Switzerland ^2^ Institute of Anesthesiology, Institute of Anesthesiology, University of Zurich, University Hospital Zurich, Zurich, Switzerland ^3^ Department of Pathology, Cantonal Hospital Winterthur, Zurich, Switzerland
^4^ Division of Digestive Diseases, Rush University Medical Center, Chicago, Illinois, USA
^5^ Team Pathogenesis of viral hepatitis UMR INSERM 1052 - CNRS 5286 Centre de recherche en cancerologie de Lyon, France
^6^ Romain Parent Inserm U1052 Team #15 - Lyon Cancer Research Center, France
^7^ Department of Surgery, Division of Transplant Surgery, Rush University Medical Center, Chicago, Illinois, USA
^8^ Department of Surgery, Cantonal Hospital Winterthur, Zurich, Switzerland
^9^ Department of Anesthesiology, University of Illinois at Chicago, Chicago, Illinois, USA

^*^ corresponding author

**Correspondance**
Erik Schadde, MD FACS FEBS (HPB)
Institute of Physiology
University of Zürich
Winterthurerstr. 190
CH-8057 Zürich
Phone: +41 79 418 24 45
Email: [erik.schadde@uzh.ch](mailto:erik.schadde@uzh.ch)

| **Supplementary Table 1: Differently expressed transcripts after 24 hours treatment of LX-2 cells with 1 mM DMOG Functional annotation using the Database for Annotation, Visualization, and Integrated Discovery (DAVID)** | | | |
| --- | --- | --- | --- |
| Symbol | Gene Name | Fold Change | p-value |
| **HIF-1 pathway** | |  |  |
| UP-REGULATED | | | |
| PFKFB3 | 6-phosphofructo-2-kinase/fructose-2,6-biphosphatase 3 | 4.12 | 0.0007 |
| CAMK2D | calcium/calmodulin-dependent protein kinase II delta | 3.97 | 0.00008 |
| CDKN1A | cyclin-dependent kinase inhibitor 1A (p21, Cip1) | 7.32 | 0.0004 |
| EDN1 | Endothelin 1 | 3.89 | 0.0024 |
| EGFR | epidermal growth factor receptor | 2.32 | 0.0011 |
| FLT1 | fms-related tyrosine kinase 1 | 4.56 | 0.0038 |
| HK2 | hexokinase 2 | 10.21 | 0.00004 |
| PDK1 | pyruvate dehydrogenase kinase, isozyme 1 | 3.02 | 0.0002 |
| SLC2A1 | solute carrier family 2 (facilitated glucose transporter), member 1 | 3.27 | 0.0000006 |
| DOWN-REGULATED | | | |
| PIK3CB | phosphatidylinositol-4,5-bisphosphate 3-kinase, catalytic subunit beta | -2.16 | 0.0001 |
| PIK3CG | phosphatidylinositol-4,5-bisphosphate 3-kinase, catalytic subunit gamma | -3.41 | 0.0046 |
| TLR4 | toll-like receptor 4 | -10.1 | 0.000006 |
| **Angiogenesis** | |  |  |
| UP-REGULATED | | | |
| ANGPTL4 | angiopoietin like 4 | 3.25 | 0.0055 |
| ECM1 | extracellular matrix protein 1 | 2.31 | 0.0004 |
| FLT1 | fms-related tyrosine kinase 1 | 4.56 | 0.0038 |
| HSPG2 | heparan sulfate proteoglycan 2 | 2.49 | 0.0067 |
| NRXN3 | neurexin 3 | 3.17 | 0.0015 |
| RHOB | ras homolog family member B | 2.36 | 0.002 |
| SEMA3E | sema domain, immunoglobulin domain (Ig), short basic domain, secreted, (semaphorin) 3E | 3.86 | 0.0031 |
| DOWN-REGULATED | | | |
| ARHGAP22 | Rho GTPase activating protein 22 | -2.57 | 0.0022 |
| ARHGAP24 | Rho GTPase activating protein 24 | -2.59 | 0.0014 |
| AGGF1 | angiogenic factor with G-patch and FHA domains 1 | -3.1 | 0.0007 |
| CCBE1 | collagen and calcium binding EGF domains 1 | -2 | 0.0054 |
| COL8A1 | collagen, type VIII, alpha 1 | -2.66 | 0.002 |
| C1GALT1 | C1GALT1 specific chaperone 1 | -2.67 | 0.0039 |
| ESM1 | endothelial cell-specific molecule 1 | -2.97 | 0.0037 |
| EFNB2 | ephrin-B2 | -2.48 | 0.003 |
| NRP1 | neuropilin 1 | -2.52 | 0.00001 |
| PIK3CG | phosphatidylinositol-4,5-bisphosphate 3-kinase, catalytic subunit gamma | -3.41 | 0.0046 |
| PDCD6 | programmed cell death 6 | -2.52 | 0.0007 |
| ZNF304 | zinc finger protein 304 | -2.82 | 0.002 |
| **VEGF signaling pathway** | |  |  |
| UP-REGULATED | | | |
| VEGFA | vascular endothelial growth factor A | 2.59 | 0.00007 |
| CASP9 | caspase 9 | 7.37 | 0.00002 |
| NFATC2 | nuclear factor of activated T-cells, cytoplasmic, calcineurin-dependent 2 | 14.15 | 0.00007 |
| PPP3C | protein phosphatase 3, catalytic subunit, gamma isozyme | 2.04 | 0.0048 |
| DOWN-REGULATED | | | |
| MAPKAPK3 | mitogen-activated protein kinase-activated protein kinase 3 | -2.32 | 0.0007 |
| PIK3CB | phosphatidylinositol-4,5-bisphosphate 3-kinase, catalytic subunit beta | -2.16 | 0.0001 |
| **Apoptosis** | |  |  |
| UP-REGULATED | | | |
| CASP9 | caspase 9 | 7.37 | 0.00002 |
| CASP2 | caspase 2 | 2.22 | 0.003 |
| NFKBIA | nuclear factor of kappa light polypeptide gene enhancer in B-cells inhibitor, alpha | 2.35 | 0.003 |
| TNFAIP3 | tumor necrosis factor, alpha-induced protein 3 | 3.88 | 0.00009 |
| TNFRSF10D | tumor necrosis factor receptor superfamily, member 10d, decoy with truncated death domain | 2.25 | 0.0028 |
| FAF1 | Fas (TNFRSF6) associated factor 1 | 2.95 | 0.00002 |
| WWOX | WW domain containing oxidoreductase | 2.03 | 0.0004 |
| BAG1 | BCL2-associated athanogene | 2.72 | 0.0004 |
| BAG3 | BCL2-associated athanogene 3 | 2.72 | 0.00004 |
| GADD45B | growth arrest and DNA-damage-inducible, beta | 5.27 | 0.0001 |
| BRE | brain and reproductive organ-expressed (TNFRSF1A modulator) | 2.24 | 0.0001 |
| ACVR1C | activin A receptor type IC | 2.49 | 0.007 |
| BNIP1 | BCL2/adenovirus E1B 19kDa interacting protein 1 | 2.34 | 0.0028 |
| CSRNP1 | cysteine-serine-rich nuclear protein 1 | 5.57 | 0.0005 |
| CSRNP2 | cysteine-serine-rich nuclear protein 2 | 2.77 | 0.0022 |
| DDIT4 | DNA damage inducible transcript 4 | 3.52 | 0.0003 |
| ERN1 | endoplasmic reticulum to nucleus signaling 1 | 5.09 | 0.0002 |
| BCL2A1 | BCL2-related protein A1 | 2.97 | 0.0049 |
| HIPK3 | homeodomain interacting protein kinase 3 | 3.76 | 0.0001 |
| KLF11 | Kruppel-like factor 11 | 3.48 | 0.0011 |
| PIM1 | Pim-1 proto-oncogene, serine/threonine kinase | 4.07 | 0.0002 |
| MEF2A | myocyte enhancer factor 2A | 2.36 | 0.0008 |
| IGFBP3 | insulin like growth factor binding protein 3 | 4.84 | 0.0006 |
| RYBP | RING1 and YY1 binding protein | 5.98 | 0.0002 |
| PEG10 | paternally expressed 10 | 2.14 | 0.0057 |
| SH3KBP1 | SH3-domain kinase binding protein 1 | 2.15 | 0.0004 |
| PPIF | peptidylprolyl isomerase F | 2.15 | 0.0044 |
| SHF | Src homology 2 domain containing F | 2.42 | 0.0055 |
| PRKCD | protein kinase C, delta | 2.1 | 0.0063 |
| PPP1R15A | protein phosphatase 1, regulatory subunit 15A | 3.99 | 0.0002 |
| PPP2R2B | protein phosphatase 2, regulatory subunit B, beta | 3.41 | 0.0033 |
| RHOB | ras homolog family member B | 2.36 | 0.002 |
| SIAH2 | siah E3 ubiquitin protein ligase 2 | 2.82 | 0.0002 |
| DOWN-REGULATED | | | |
| CASP8AP2 | caspase 8 associated protein 2 | -3.24 | 0.00008 |
| CASP8 | caspase 8, apoptosis-related cysteine peptidase | -5.02 | 0.0002 |
| CASP6 | caspase 6 | -2.15 | 0.0057 |
| APIP | APAF1 interacting protein | -2.96 | 0.00005 |
| BLID | BH3-like motif containing, cell death inducer; mir-100-let-7a-2 cluster host gene | -3.09 | 0.0006 |
| CHAC1 | ChaC glutathione-specific gamma-glutamylcyclotransferase 1 | -2.77 | 0.0012 |
| DNAJA3 | DnaJ (Hsp40) homolog, subfamily A, member 3 | -2.01 | 0.0031 |
| GRAMD4 | GRAM domain containing 4 | -2.12 | 0.0057 |
| HTRA2 | HtrA serine peptidase 2 | -2.97 | 0.00008 |
| NME6 | NME/NM23 nucleoside diphosphate kinase 6 | -4.15 | 0.0017 |
| PRELID1 | PRELI domain containing 1 | -2.07 | 0.0002 |
| LMNB1 | lamin B1 | -2.14 | 0.001 |
| PARP1 | poly(ADP-ribose) polymerase 1 | -3.74 | 0.00007 |
| DFFA | DNA fragmentation factor, 45kDa, alpha polypeptide | -2.35 | 0.00003 |
| FADD | Fas (TNFRSF6)-associated via death domain | -5.41 | 0.0021 |
| PIK3CB | phosphatidylinositol-4,5-bisphosphate 3-kinase, catalytic subunit beta | -2.16 | 0.0001 |
| PIK3CG | phosphatidylinositol-4,5-bisphosphate 3-kinase, catalytic subunit gamma | -3.41 | 0.0046 |
| TLN1 | talin 1; microRNA 6852 | -2.14 | 0.00004 |
| TNFAIP8 | tumor necrosis factor, alpha-induced protein 8 | -2.92 | 0.0001 |
| WDR92 | WD repeat domain 92 | -3.24 | 0.00005 |
| AIMP2 | aminoacyl tRNA synthetase complex-interacting multifunctional protein 2 | -2.74 | 0.0017 |
| CARD6 | caspase recruitment domain family, member 6 | -3.79 | 0.0008 |
| C3orf38 | chromosome 3 open reading frame 38 | -2.01 | 0.0066 |
| C6orf120 | chromosome 6 open reading frame 120 | -6.04 | 0.0006 |
| DIDO1 | death inducer-obliterator 1 | -3.78 | 0.0003 |
| EBAG9 | estrogen receptor binding site associated, antigen, 9 | -2.47 | 0.00005 |
| MFSD10 | major facilitator superfamily domain containing 10 | -2.22 | 0.0031 |
| MOAP1 | modulator of apoptosis 1 | -3.77 | 0.0016 |
| PPID | peptidylprolyl isomerase D | -3.11 | 0.00007 |
| PDCD2 | programmed cell death 2 | -2.13 | 0.0005 |
| PDCD4 | programmed cell death 4 (neoplastic transformation inhibitor); microRNA 4680 | -2.29 | 0.0038 |
| PDCD6 | programmed cell death 6 | -2.52 | 0.0007 |
| PDCD7 | programmed cell death 7 | -5.32 | 0.00005 |
| PSMD10 | proteasome 26S subunit, non-ATPase 10 | -2.41 | 0.0003 |
| RNF34 | ring finger protein 34, E3 ubiquitin protein ligase | -3.21 | 0.0039 |
| RNF41 | ring finger protein 41, E3 ubiquitin protein ligase | -2.12 | 0.0029 |
| STPG1 | sperm-tail PG-rich repeat containing 1 | -3.34 | 0.0001 |
| TMBIM4 | transmembrane BAX inhibitor motif containing 4 | -2.24 | 0.0008 |
| TRIM69 | tripartite motif containing 69 | -2.5 | 0.0007 |
| ZC3H8 | zinc finger CCCH-type containing 8 | -2.15 | 0.0017 |
| **p53 pathway** | |  |  |
| UP-REGULATED | | | |
| CASP9 | caspase 9 | 7.37 | 0.00002 |
| CCNG2 | cyclin G2 | 2.34 | 0.0036 |
| CDK6 | cyclin-dependent kinase 6 | 2.37 | 0.0077 |
| CDKN1A | cyclin-dependent kinase inhibitor 1A (p21, Cip1) | 7.32 | 0.0004 |
| GADD45A | growth arrest and DNA-damage-inducible, alpha | 2.33 | 0.0024 |
| GADD45B | growth arrest and DNA-damage-inducible, beta | 5.27 | 0.0001 |
| DOWN-REGULATED | | | |
| CASP8 | caspase 8, apoptosis-related cysteine peptidase | -5.02 | 0.0002 |
| CCNB2 | cyclin B2 | -2.22 | 0.0003 |
| RCHY1 | ring finger and CHY zinc finger domain containing 1, E3 ubiquitin protein ligase | -3.56 | 0.0012 |
| SESN1 | sestrin 1 | -2.01 | 0.0032 |
| **ECM-ECM-receptor-interaction** | |  |  |
| UP-REGULATED | | | |
| CCL20 | chemokine (C-C motif) ligand 20 | 5.89 | 0.0015 |
| CCL5 | chemokine (C-C motif) ligand 5 | 4.31 | 0.0055 |
| CXCL12 | chemokine (C-X-C motif) ligand 12 | 2.41 | 0.0005 |
| CXCL8 | chemokine (C-X-C motif) ligand 8 | 6.03 | 0.0002 |
| ACKR3 | atypical chemokine receptor 3 | 12.4 | 0.0003 |
| CLCF1 | cardiotrophin-like cytokine factor 1 | 3.12 | 0.0055 |
| CNTF | ciliary neurotrophic factor | 2.07 | 0.0065 |
| CCDC80 | coiled-coil domain containing 80; long intergenic non-protein coding RNA 1279 | 2.92 | 0.0003 |
| EGFR | epidermal growth factor receptor | 2.32 | 0.0011 |
| FLT1 | fms-related tyrosine kinase 1 | 4.56 | 0.0038 |
| IL11 | interleukin 11 | 4.77 | 0.005 |
| LIF | leukemia inhibitory factor | 2.84 | 0.0002 |
| SPOCK1 | sparc/osteonectin, cwcv and kazal-like domains proteoglycan (testican) 1 | 2.47 | 0.0036 |
| ANGPTL4 | angiopoietin like 4 | 3.25 | 0.0055 |
| BGN | biglycan | 2.21 | 0.0087 |
| CPZ; GPR78 | carboxypeptidase Z; G protein-coupled receptor 78 | 2.28 | 0.0001 |
| COL5A3 | collagen, type V, alpha 3 | 2.05 | 0.01 |
| COL12A1 | collagen, type XII, alpha 1 | 2.75 | 0.0063 |
| ECM1 | extracellular matrix protein 1 | 2.31 | 0.0004 |
| FBN1 | fibrillin 2 | 2.02 | 0.0002 |
| FBN2 | fibrillin 2 | 2.06 | 0.0078 |
| HSPG2 | heparan sulfate proteoglycan 2 | 2.49 | 0.0067 |
| LAMA1 | laminin, alpha 1 | 3.05 | 0.0002 |
| LAMA3 | laminin, alpha 3 | 2.06 | 0.0042 |
| MMP1 | matrix metallopeptidase 1 | 5.04 | 0.0055 |
| MMP16 | matrix metallopeptidase 16 (membrane-inserted) | 2.48 | 0.0091 |
| POSTN | periostin, osteoblast specific factor | 4.09 | 0.0002 |
| PODN | podocan | 3.98 | 0.0041 |
| TNC | tenascin C | 6.48 | 0.0002 |
| VCAN | versican | 3.87 | 0.0028 |
| DOWN-REGULATED | | | |
| COL4A5 | collagen, type IV, alpha 5 | -2.24 | 0.0028 |
| COL4A3BP | collagen, type IV, alpha 3 (Goodpasture antigen) binding protein | -3.04 | 0.0002 |
| COL6A3 | collagen, type VI, alpha 3 | -2.66 | 0.0055 |
| COL8A1 | collagen, type VIII, alpha 1 | -2.66 | 0.002 |
| ITGA2 | integrin, alpha 2 (CD49B, alpha 2 subunit of VLA-2 receptor) | -3.5 | 0.00005 |
| ITGA5 | integrin alpha 5 | -2.14 | 0.00007 |
| ITGB5 | integrin beta 5 | -4.49 | 0.0003 |
| ITGBL1 | integrin beta like 1 | -2.83 | 0.0062 |
| CCBE1 | collagen and calcium binding EGF domains 1 | -2 | 0.0054 |
| ADAMTS7 | ADAM metallopeptidase with thrombospondin type 1 motif 7 | -3.14 | 0.0021 |
| BMP4 | bone morphogenetic protein 4 | -5.99 | 0.0001 |
| MFAP1 | microfibrillar associated protein 1 | -3.46 | 0.0001 |
| NTN4 | netrin 4 | -2.75 | 0.0012 |
| NID2 | nidogen 2 (osteonidogen) | -3.65 | 0.0033 |
| P3H1 | prolyl 3-hydroxylase 1 | -3.38 | 0.0002 |
| LRP5 | LDL receptor related protein 5 | -2.62 | 0.0072 |
| CCBE1 | collagen and calcium binding EGF domains 1 | -2 | 0.0054 |

| **Supplementary Table 2: Differently expressed transcripts of TRP3 cells after 24 hours incubation with conditioned medium of DMOG-treated LX-2 cells Functional annotation using the Database for Annotation, Visualization, and Integrated Discovery (DAVID)** | | | |
| --- | --- | --- | --- |
| Symbol | Gene Name | Fold Change | p-value |
| **HIF-1 pathway** | |  |  |
| UP-REGULATED | | | |
| VEGFA | vascular endothelial growth factor A | 7.74 | 0.003 |
| ANGPT2 | angiopoietin 2 | 5.04 | 0.0004 |
| EGLN1 | egl-9 family hypoxia-inducible factor 1 | 2.93 | 0.0055 |
| EGLN3 | egl-9 family hypoxia-inducible factor 3 (↑↑↑) | 35.85 | 0.0001 |
| ENO2 | enolase 2 (gamma, neuronal) | 2.47 | 0.0006 |
| SLC2A1 | solute carrier family 2 (facilitated glucose transporter), member 1 | 4.61 | 0.00003 |
| **Angiogenesis** | |  |  |
| UP-REGULATED | | | |
| SHB | Src homology 2 domain containing adaptor protein B | 2.1 | 0.00006 |
| ANGPT2 | angiopoietin 2 | 5.04 | 0.0004 |
| ANGPTL4 | angiopoietin like 4 | 13.44 | 0.0024 |
| DLL4 | delta-like 4 (Drosophila) | 17.87 | 0.0063 |
| EFNA1 | ephrin-A1 | 2.92 | 0.0009 |
| PGF | placental growth factor | 2.34 | 0.0011 |
| **Apoptosis** | |  |  |
| UP-REGULATED | | | |
| RASSF2 | Ras association (RalGDS/AF-6) domain family member 2 | 2.55 | 0.0017 |
| CCNY | cyclin Y | 2.42 | 0.0021 |
| DUSP1 | dual specificity phosphatase 1 | 4.49 | 0.0023 |
| RGCC | regulator of cell cycle | 20.12 | 0.0008 |
| SIK1 | salt-inducible kinase 1 | 3.55 | 0.0003 |
| SNX33 | sorting nexin 33 | 3.87 | 0.0003 |
| ZFYVE19 | zinc finger, FYVE domain containing 19 | 2.13 | 0.0038 |
| SHANK3 | SH3 and multiple ankyrin repeat domains 3 | 2.37 | 0.0053 |
| WDR60 | WD repeat domain 60 | 2.05 | 0.0002 |
| ATL1 | atlastin GTPase 1 | 2.46 | 0.0068 |
| PODXL | podocalyxin-like | 2.35 | 0.0002 |
| KCNIP1 | Kv channel interacting protein 1 | 3.7 | 0.0017 |
| SPAG4 | sperm associated antigen 4 | 2.91 | 0.0005 |
| SHB | Src homology 2 domain containing adaptor protein B | 2.1 | 0.00006 |
| EGLN3 | egl-9 family hypoxia-inducible factor 3 (↑↑↑) | 35.85 | 0.0001 |
| ERO1A | endoplasmic reticulum oxidoreductase alpha | 2.59 | 0.0057 |
| IGFBP3 | insulin like growth factor binding protein 3 | 7.63 | 0.0007 |
| ANGPT2 | angiopoietin 2 | 5.04 | 0.0004 |
| ANGPTL4 | angiopoietin like 4 | 13.44 | 0.0024 |
| DLL4 | delta-like 4 (Drosophila) | 17.87 | 0.0063 |
| KDM3A | lysine (K)-specific demethylase 3A | 2.75 | 0.0018 |
| PGF | placental growth factor | 2.34 | 0.0011 |
| SFRP1 | secreted frizzled-related protein 1 | 2.01 | 0.0029 |
| SLC2A14 | solute carrier family 2 (facilitated glucose transporter), member 14 | 3.26 | 0.0001 |
| DOWN-REGULATED | | | |
| CDKN2A | cyclin-dependent kinase inhibitor 2A | -2.4 | 0.0096 |
| MCM4 | minichromosome maintenance complex component 4 | -5.04 | 0.0053 |
| MCM8 | minichromosome maintenance 8 homologous recombination repair factor | -2.41 | 0.0051 |
| PCNA | proliferating cell nuclear antigen | -2.85 | 0.0035 |
| CLSPN | claspin | -2.04 | 0.0032 |
| RCC2 | regulator of chromosome condensation 2 | -2.05 | 0.0031 |
| BMX | BMX non-receptor tyrosine kinase | -4.1 | 0.0004 |
| TNFRSF1B | tumor necrosis factor receptor superfamily, member 1B; microRNA 4632; microRNA 7846 | -2.73 | 0.0003 |
| TNFSF10 | tumor necrosis factor (ligand) superfamily, member 10 | -16.15 | 0.0003 |
| HMOX1 | heme oxygenase 1 | -2.89 | 0.0029 |
| AHI1 | Abelson helper integration site 1 | -2.09 | 0.0027 |
| NAA15 | N(alpha)-acetyltransferase 15, NatA auxiliary subunit | -2.51 | 0.000004 |
| CFAP54 | cilia and flagella associated 54 | -2.21 | 0.0081 |
| LGR4 | leucine-rich repeat containing G protein-coupled receptor 4 | -2.85 | 0.003 |
| SPATA5 | spermatogenesis associated 5 | -2.06 | 0.001 |
| SIGMAR1 | sigma non-opioid intracellular receptor 1 | -2.83 | 0.0004 |
| SORD | sorbitol dehydrogenase | -2.94 | 0.0032 |
| SYNJ2 | synaptojanin 2 | -29 | 0.00003 |
